# “Patients would probably be more compliant to therapy if encouraged by those around them”: a qualitative study exploring primary care physicians’ perceptions on barriers to CVD risk management

**DOI:** 10.1186/s12875-022-01668-0

**Published:** 2022-03-30

**Authors:** Nikansha Kumar, Masoud Mohammadnezhad

**Affiliations:** School of Public Health and Primary Care, Fiji National University, Suva, Fiji

**Keywords:** Physicians, Perceptions, Barriers, Cardiovascular diseases, Risk management, Management, Fiji

## Abstract

**Background:**

Public health prevention strategies have been developed to overcome the disease burden. Despite all the available resources, there have been several challenges in keeping up with the practices in Cardiovascular Diseases (CVD) risk management. The aim of this study is to explore Primary Care Physicians’ (PCP) perceptions on barriers to CVD risk management and strategies to overcome these barriers in Suva, Fiji.

**Methods:**

This is a qualitative study conducted in the Suva Medical area among 7 health centers from August 1st to September 30th, 2021. Purposive sampling was used to recruit participants who provided in-depth and detailed information. From those physicians who worked in the Suva medical area as Primary Health Care (PHC) physicians, those who had at least 6 months experience and those who had some Special Outpatients Department (SOPD) exposure, in-depth interview was conducted using semi-structured questionnaire over the telephone and recorded in a mobile app. The interview content was then transcribed and thematic analysis was done.

**Results:**

This study included 25 PHC physicians. From the thematic analysis, 2 major themes were developed including perceived barriers to CVD risk management and some of the strategies to overcome these barriers. Some of the barriers identified include patient factors such as non-compliance, physician factors such as time-constraints and lack of training, and health system factors such as poor medical records system and lack of basic resource. The strategic support systems include patient expectations, physician’s encouragement, utilization of resources, laws and legislations and continuing evidence-based medicine.

**Conclusions:**

Physicians’ perceptions on the barriers and the strategies to overcome those barriers in CVD risk management plays an important role. The barriers include those of physician factors, patient factors and the health system as a whole. It is suggested to encourage shared-decision making in CVD management, enhance physician support and reinforce policies and research to bring about positive change and improvements in the quality of care.

## Background

Cardiovascular Diseases (CVDs) are the leading causes of death and disability worldwide despite the availability of well-established and effective preventive options and information. Public health prevention strategies have been developed to overcome the disease burden [1, 2]. The World Health Organization (WHO) provides back-up to the governments especially in Low- and Middle- Income Countries (LMICs) in preventing, managing and monitoring of CVDs by providing global health strategies to decrease risk factors, enhance standards of care, improve capacity of health system in care of patients, and observing the trends and disease patterns [1, 3].

Fiji is a Pacific Island nation with a population of approximately 889,327 comprising of multiracial groups including I-Taukei, Fijians of Indian decent and others (Asians and Europeans) [4]. The World Bank classifies Fiji as an upper-middle income country [5]. The health system in Fiji is established on a three-tier model of primary, secondary and tertiary levels and has made significant progress in its field based on the Millennium Development Goals (MDGs) [6]. The health system includes both the public and private sectors. The primary health care system is decentralized and the primary health care services are provided by both the private and public health sectors [6].

Fiji has experienced epidemiological changeover whereby childhood mortality and infectious diseases are declining and the mortality from CVDs are peaking [7]. Fiji had almost double CVD related deaths in 2017 [8]. This was significant with the global trends [9]. The Pacific Islands leaders have declared the Pacific region being in Non-Communicable Diseases (NCDs) ‘crisis’. It is estimated that about 60% of death in Solomon Islands and 77% in Fiji are due to NCDs. NCDs have led to premature deaths in the Pacific which is comparable to LMICs [10].

The Fiji urban population have access to both the public and private medical care, however for rural settings, majority have access to public health care [6]. The public health care is easily accessible and available at free of cost in Fiji [11]. With majority of the population reaching out to the public health sectors for medical services, there have been many challenges faced in the primary health care system. Developed countries may cope with these challenges however, with the developing countries, strategies need to be in place to handle the barriers and challenges [12].

Despite physicians’ awareness on CVDs and prevention goals at primary and secondary levels and the prospective to improve clinical outcomes with the lifestyle modifications and the guidelines and treatments, the application of these strategies if far from ideal [13–15]. The World Bank report on “Pacific Islands: Non-Communicable Disease Roadmap” states that there are four main approaches that all Pacific countries could strategize. These include health food consumption with low salt, low sugar and low fat contents of food, reinforcing the tobacco control laws and regulations, enlightenment on the prevention and early treatment strategies and strengthening the monitoring and evaluation of the implemented programs. These approaches have been envisioned to be cost-effective and achievable however requires utter determination and leadership. In this perspective, physicians play a vital role in primary health care thus it is crucial to take into consideration their viewpoints in CVD management [16].

Little is known about what the primary care physicians perceive as barriers to CVD risk management especially in LMICs and there are no available data on the perceptions of physicians in Fiji. Thus, the aim of this study is to explore primary care physicians’ perceptions on the barriers to CVD risk management and strategies to overcome these barriers in Suva, Fiji.

## Methods

### Study design and setting

This study applied a qualitative study design using in-depth interviews via phone calls to explore the primary care physicians’ perceptions on barriers to CVD risk management and the strategies to overcome these barriers. This study was conducted from August 1st to September 30th, 2021, in the Suva Medical area which includes Lami Health Centre (HC), Samabula HC, Nuffield HC, Raiwaqa HC, Makoi HC, Valelevu HC and Nakasi HC. These 7 Health Centers provide primary health care to about 225,856 people and approximately 50 doctors are employed across these health centers. The public health sector was chosen due to the vast number of patients seeking primary health care under the Government’s initiative to provide free medical care to the public.

### Study sample

All doctors working in the public health centers in the Suva medical area were invited to participate in the study. This study included those who worked in Suva medical area as a PHC doctor for at least 6 months and have exposure in conducting Special Outpatients Clinic (SOPD) clinic. Doctors who worked in the private sector only, locum doctors working in health centres and doctors who were not willing to participate were excluded. The selection was based on the fact that many of the general public seek primary health care in Government health facilities. This is on the basis that many of the public health physicians are trained in the WHO based guidelines, many of the physicians work closely within the community to provide primary care services and many of these physicians continue to work in public health sectors. In addition, majority of the population are followed-up for their general health conditions including NCDs at the SOPD clinics at their nearest health centres. Patients who are no longer able to afford the private sector treatment plans are also referred back to their decentralized health centres for further management.

Purposive sampling was used to recruit participants whereby researcher recruited participants who could provide in-depth and detailed information about the aim of the investigation. In this study, 3 – 4 primary health care physicians from each health center, who met the criteria, were chosen for in-depth interviews. Overall, 25 medical officers participated in this study. At 20 participants, data saturation was attained however, 5 more were included to ensure no important point was missed out.

### Data collection tool

A semi-structured open-ended questionnaire was used to guide the interviews. This questionnaire was developed based on literature review and the aim and research questions of the study. The questionnaire included 2 sections; Section 1 includes demographics and general information such as participant’s age, gender and work experience while the second section included 6–7 open ended questions to understand their perceptions on CVDs risk management. Probing questions were added based on the response from the interviewee. Before using this questionnaire, the content of the questionnaire was assessed by 3 experts.

### Study procedure

The participants were notified of the study through the Medical Officer-In-Charge (MOIC) at the various health facilities. Invitation letters were sent to all medical officers 1 month prior in the Suva Medical area to participate in the study. Those who met the inclusion criteria and had consented to take part in the study were short-listed for participation in the in-depth interview. A convenient time was arranged with the participants based on their shift of work. Participant information sheet and consent forms were distributed to the participants. Participating officers were interviewed via telephone. The telephone contacts were obtained through the MOIC at the respective health centers. This was in consideration of Fiji’s current battle with a global pandemic, Coronavirus Disease of 2019 (COVID-19). The Medical-Officers were informed on the interview time and date, and as per their convenience, data was collected. The total time was approximately 30 – 40 min per participant. The interview was recorded on sound recorder app in a tablet device.

### Data analysis

All interview recordings was transcribed verbatim by the principal researcher and reviewed by the co-researcher. The data was analyzed using manual thematic analysis. Thematic analysis is used in qualitative data analysis. In thematic analysis, the researcher looks at the data closely and finds similar or common themes including topics, meanings and ideas that appear frequently [17].

Thematic analysis followed an interactive approach, whereby as new themes were identified and added to the thematic framework, earlier transcripts were re-coded. Later both data were compared, contrasted and interpreted to develop a conceptual matrix thematic analysis, whereby themes are generated from the data as opposed to a pre-existing thematic framework, was performed concurrently.

### Ethical consideration

Ethical approval to conduct this study was obtained from Fiji National University’s (FNU) College Health Research Ethics Committee (CHREC). Approval to conduct the study at the health facilities was obtained from the Ministry of Health and Medical Sciences (MoHMS), mainly from the Divisional Medical Officer (DMO). Written informed consent with rights to withdraw without any consequences, was taken from the participating doctors before collecting data and assurance of confidentiality and anonymity was provided to them throughout the course of the study and afterwards as well. All methods were carried out in accordance with relevant guidelines and regulations.

## Results

### Characteristics of the participant

Twenty five (25) medical officers participated in this study. Majority of participants (76%) were at the age category of 25 to 34 years. The gender distribution shows 5 (20%) were male participants and 20 (80%) were female participants. Based on ethnicity, 5 (20%) were I-Taukei, [18] (72%) were Fijians of Indian decent and 2 (8%) were from other ethnic category. Majority of participants (52%) had less than 5 years’ experience while only 12% had more than 16 years’ experience.

Out of the 25, 22 participants were Fiji Island graduates and the other 3 graduated from other countries and were employed here in Fiji with full license. Two physicians have Master of Public Health degree, 4 have a Post-Graduate Diploma in NCDs and the remaining 19 have only the Bachelor of Medicine and Bachelor of Surgery (under-graduate) degree (Table 1).

**Table 1:** Characteristics of participants (*N* = 25)

Characteristics	n (%)
**Age (yrs.)**	
25 – 34	19 (76)
35 – 44	4 (16)
45 – 54	1 (4)
> 55yrs	1 (4)
**Gender**	
Male	5 (20)
Female	20 (80)
**Ethnicity**	
I-Taukei	5 (20)
Indo-Fijian	18 (72)
Others	2 (8)
**Years of experience (yrs.)**	
0 – 5	13 (52)
6 – 10	7 (28)
11 – 15	2 (8)
16 and over	3 (12)
**Place of tertiary studies**	
Fiji Islands	22
Outside Fiji	3
**Tertiary Qualification**	
Master of Public Health	2
Post-graduate diploma in NCDs	4
MBBS – Bachelor of Medicine and Bachelor of Surgery	19

### Themes and subthemes identified

Two major themes were developed with 8 subthemes. Theme 1 was perceived barriers to CVD risk management and Theme 2 as strategies to overcome these barriers. Table 2 shows the themes and sub-themes identified from the participants’ interview. The responses from the participants are quoted below with ‘FD’ defined as ‘female doctor’ and ‘MD’ defined as ‘male doctor’.

**Table 2:** Themes and sub-themes identified from interview

Themes	Subthemes
**Perceived barriers to CVD risk management**	Physician-related factors
	Patient-related factors
	Health system-related factors
**Strategies to overcome these barriers**	Patient expectation and outcome
	Physicians encouragement and support
	Resources
	Laws, legislations and policies
	Ongoing evidence – based medicine

### Theme 1: perceived barriers to CVD risk reduction

Theme 1 include 3 subthemes; physician factors, patient factors and health system factors that act as barriers to CVD risk management.

### Physician factors

The participants listed various physician related factors as potential barriers in CVD risk management. Most believed that it’s a combination of factors that become a barrier and not just a single factor.

“Its multi-factorial I guess. Some of the doctor related issues I can think of are level of training and experience in this field, availability of the clinical practice guidelines and assessment tools and then our ability to correctly use these resources play an important part. The support of our other colleagues and Ministry in provision of appropriate tools are also important factors. Our personal attributes like how involved we are towards our work and confidence in handling a wide spectrum of cases also contribute” (30 years old FD)

A participant mentioned access to guidelines as a barrier:

“Yeah, we don’t have it available to us in written or in hard copy so most of the time I have to use my phone and it that sense, I am always on the phone and patients think that I am not doing work and I am doing personal work.” (32 years old MD)

Another participant highlighted on “doctor shopping”:

“…I have had patients elderly i-Taukei patients who prefer an i-Taukei doctor, in those situations I don’t really mind because I do understand that they want to speak to someone who would be able to communicate with them in their own language, so that they can understand easily what they trying to say…but sometimes, yes, we do get patients who are not happy with one doctor’s opinion so they go to another doctor to seek their opinion…so there’s no track to see what these patients are doing, for example they go to a private doctor, they prescribe something and then they come to GOPD or SOPD and then they are prescribed something…” (27 years old FD)

### Patient factors

Participants identified the various patient related factors as barriers to CVD risk assessment and management. Almost all participants highlighted patient compliance an issue.

“Patient compliance is an issue so patients are not compliant. They have many reasons for this…one thing could be their socio-economic status, another thing is their hesitance to actually take drugs, medications and then their hearsay, what they hear from their neighbors or on social media, the un-research or under-researched treatment alternatives that they are all trying because someone else is trying…so those are like difficulties that we actually face…and also if patients don’t understand then they do not follow…understanding of the disease…the stigma.” (36 years old FD)

Another patient highlighted education and social background as important barriers.

Patients’ education levels, their socio-economic status, family support and accessibility to health care are some of the factors. Another issue is when it comes to the mind set of patients. Especially when they are in denial or if they have the fear of being judged or they don’t have the will power to change their behavior and lifestyle.” (29 years old FD)

Another participant mentioned about financial constraints and mental health issues.

“Well from the patients’ perspective, mostly, I would say availability of medications, and then coming to the facility, sometimes the financial constraints…mental status for the newly diagnosed or the young patients.” (34 years old FD)

### Health care system factors

Participants also identified healthcare system related factors as barriers to CVD risk assessment and management.

“One of the issues here is lack of resources. We don’t have enough manpower…space-wise, we do not have a special SOPD area in our health center. So lack of resources is one of those problems and also medications are not available free…most of time and often patients are not able to purchase the medications and as a result they are not able to adhere to the medications.” (36 years old FD)

A participant highlighted on availability of medications:

“Not much of a difficulty when diagnosing, but yes, with management there are difficulties. Availability of resources or drugs. Most of the medications are out of stock in our government pharmacies. This can be directly related to drug compliance and halts management.” (29 years old FD)

Few participants also highlighted delays in obtaining laboratory results:

“Diagnosing is ok but…for example if I want to diagnose a patient for diabetes, then we have to send to for bloods like HbA1c for confirmation and Hba1c is not readily available, most of the time, so that is another challenge that I face. And for hypertension for example, we have to call patients for review few times and book them for motivational interview.” (34 years old FD)

Another participants stated on the need for proper medical record system.

“…We need to digitalize our medical record system. PATIS should be reinforced. Patient records and data should be made easily accessible so all doctors have access to those information. For instance, the patients who visit both the private doctors and the health centers, they get medications and other treatments everywhere but are lost when trying to put things together…even the small notes on the vital information should be recorded online, so every time new doctor sees the patient he will find it easy to refer and manage appropriately.” (26 years old FD)

Doctor-patient relationship, building rapport, communication skills, access to health care on ad hoc basis, unforeseen circumstances (e.g. COVID-19) and patient-record systems are some of the others factors identified as barriers by the participants.

“The non-technical or non-clinical attributes of a doctor plays an important role.. Example their communication skills, their religious beliefs may hinder their ability to accurately assess and manage their CVD patients. This will hold true for patient as well, like how well or how poorly they communicate about their issues or their religious beliefs” (29 years old FD).

Another participant mentioned that:

“Some patients miss their clinics and might show up on days when there is no time for the doctor to see them in SOPD, so they get diverted to GOPD…this doesn’t ensure in continuation in care as GOPD is not meant to assess and manage such cases comprehensively.” (30 years old FD).

Another participant spoke on the impact of unforeseen events:

“One thing I would like to point out is how the current pandemic (COVID-19) has affected our care for CVD patients. With clinics being closed for weeks, there has been a break in follow-up and appropriate escalation or de-escalation of their care…there has been disruptions in management and follow-up…we have lost a few patients as well” (50 years old FD).

### Theme 2: strategies to overcome the barriers

The last few questions of the interview focused on ways these barriers could be dealt with. Few subthemes emerged from the responses such as patient expectation and outcome, physicians’ encouragement and support, resources, laws, legislations and policies and ongoing evidence – based medicine and advancements in CVD management.

### Patient expectation and outcome

Education of patients was highlighted by almost all participants.

“This will be very challenging. I think on-going patient education holds key here. Information needs to be communicated and then re-enforced on every subsequent encounter. Use of IEC material will help. I am not sure if our system can afford but providing some form of incentives might help especially in improving compliance” (32 years old MD)

Effective communication was also noted by most participants.

“Communication is vital. Use of vernacular language to get message across… this will need involvement of translators. Another important aspect will be involvement of family, care-givers or even the wider community like church members or work colleagues. Patients would probably be more compliant to therapy if encouraged by those around them” (28 years old FD)

The participants were asked on ways to improve patient-oriented goals.

“Patients should be engaged in formulating their management plans. This way patients will feel encouraged and take ownership of their health thus, compliance to the agreed plan will be high.” (27 years old MD)

### Physicians’ encouragement and support

These are some of the responses from the physicians on their viewpoint on the physician factors:

“There’s always a room for improvement in everything. Especially with the medical field, we have to continuously improve ourselves and train our self and acquire the knowledge because things are changing at a very fast pace now. And we can see that previously NCD was the main component.” (50 years old FD)

A participant spoke on the value of central coordination of CVD activities.

“…have a special set of qualified people to look after the NCDs and to look after the cardiovascular to after the whole or the country, for example, the wellness committee with a wellness head and also the NCD coordinator. But I feel that there should be a, on a national level, a team who should deriving the policies and trying to gather information… for everybody to be on the same page. And also to target NCDs in different groups’ example, elders, mothers’ children and young.” (34 years old FD)

A participant spoke in length on need for strengthening GOPD services as part of addressing institutional factors:

“…Strengthen the GOPD process, the current GOPD process, maybe one would be to actually change the current GOPD system, maybe have a system whereby all these patients who are coming for review of their BPs and CBGs, to have a medical officer allocated in GOPD to be handling them. It can change according to the shift or the roster. And to strengthen that part, we actually need to regularly train all the MOs or like refresher training; this can be in-house training… in-cooperate our SOPD system, GOPD system, our PEN model, WHO risk system and how you going to manage them and how often you are going to call them and the referral process to SOPD. So if we, have a SOP…we can narrow the gaps.” (36 years old FD)

Need for on-going training was also highlighted by the participants.

“The last training I had was 2 years ago…Only 2 days of crammed training was not good…it’s too much to comprehend at one time. The training should be ongoing…it’s should be transitioned as the new changes come.” (34 years old FD)

Strengthening collaboration between physicians was also highlighted by few participants.

“…collaboration amongst doctors will help improve service delivery. For example, having a social media chat group where interesting cases and challenging patients are discussed. In this way, doctors will learn from experience of others and be better equipped to handle similar cases in their own practices.” (30 years old FD)

### Resources

Once a patient has had the basic care at the primary facility, a more advanced or a specialist care may be needed. Participants were asked how accessible help from a tertiary hospital where specialists are available is.

“It’s dependent on a case by case basis. Those who need specialist care at my workplace are usually booked to be seen in specialist outreach clinics. The specialist clinic is conducted weekly. However, due to wide range of factors including cancellation of clinics, limited number of bookings, patient not turning up for clinic, etc, plenty patients do not get timely specialist consultations”. (32 years old MD)

Generally participants believed that access to timely specialist care is poor.

“I would say that it’s very poor… especially for acute cases. For example, a patient with acute diabetic complication... say DKA (diabetic keto-acidosis) will not be transferred to a tertiary hospital for specialist care on time due to unavailability of beds, transport or case not being accepted in a timely manner. All this delays appropriate care for patient as our Health Centers are not equipped to manage such acute cases” (29 years old FD)

These are some of the responses from the physicians on their viewpoint on the other factors that affect the services:

“Something needs to be done to ensure continuation of care in times of unforeseen closure of clinics like during COVID. One option would be tele-clinics…another aspect that needs attention is accessibility to health records. There needs to be significant investment in this area to ensure a standard electronic data records system. This will ensure all patient related information be available to the attending doctor at the click of a button rather than the current system where folders go missing, incomplete data entry which leads to interruption in patient management...also internet is a need in places such as primary health care facility therefore making available hotspots in all health facilities will ensure access to information and guidelines on finger tips.” (55 years old MD)

Participants’ also highlighted need to focus on non-technical aspects of CVD management.

“While a lot of focus is on the technical aspects of management, an equally vital and often neglected component is the non-technical skills of the doctor. I suggest that this will help is this regard and improve doctors’ ability in picking non-verbal cues” (27 years old FD**)**

### Laws, legislations and policies

Perceptions of physicians were also explored in terms of laws and legislations and the policies on NCDs.

“…I think the laws are there, just needs to be reinforced.” (29 years old FD)

Another participant mentioned that:

“The current policies are good but they are under-utilized. The relevant authorities need to ensure these policies needs to be enforced.” (28 years old MD)

Few participants were also of the views that stricter policies are needed to discourage people from high risk behaviours.

“Despite the current laws and legislation, the current laws and legislations concerning CVDs out data suggests that CVD numbers are rising. This to me that the current policies are not effective and needs to be reviewed and more stringent measure applied or enforced.” (32 years old MD)

Another participant commented that:

“I don’t have much comments on the policies… the policies that we have are for an ideal situation and in an ideal setting but the situation that we have within our health centers, it’s not ideal…we need to sort of bridge that gap…this can be addressed when future policies and regulations are established…the situation and the policies should correlate”. (30 years old FD)

### Ongoing evidence – based medicine

Research and studies play an important role in improving patient care and service delivery. Participants were asked on their views on the research and studies that have been done or they would want to be done in future.

“There is lots and lots of data available, however, the utilization of this data is poor. Therefore, it will make sense that we start acting on the data that is already available in order to curb the burden of CVDs. I am aware few of my colleagues who are doing research in this field and it becomes more important that we use new data as it becomes available to escalate our response.” (28 years old FD)

Another participant who agreed on value of research mentioned:

“so with evidence-based medicines, we continue to have CMEs, we continue to keep updating ourselves...it is very important because medicine is continuously changing and everyday new evidences keeps on appearing on how better manage these patients but most of these studies and research are done in developed countries, well developed countries…and if we are trying to implement it in Fiji which is a developing nation, a third world country, we need to make those adjustments so that is matches or fits or population and our situation and setting...and the resources we have available here…studies and research have a very important role.” (27 years old FD)

## Discussion

The two themes developed in this study include perceived barriers to CVD risk management and strategies to overcome those barriers. The subthemes for barriers include physician factors, patient factor and health system factors. The subthemes for strategies include patient expectation and outcome, physicians’ encouragement and support, resources, laws, legislations and policies, and on-going evidence based medicine.

In this study, physician related factors identified as potential barriers in terms of CVD risk assessment and management include lack of education and knowledge, experience, preference, attitude, availability of resources, trainings, adherence to guidelines and self-motivation and confidence. Patient related factors include education level, insight, myths and beliefs, socio-economic support, financial status, access to health care, compliance, medications, social stigma and self-motivation. Healthcare system related factors include patient-doctor ratio, availability of allied health care workers, medications (side-effects, availability and cost), investigations and tests required, multi-disciplinary approach and health promotion materials. Doctor-patient relationship, building rapport, communication skills, access to health care on ad hoc basis, unforeseen circumstances (e.g. COVID-19) and patient-record systems are some of the others factors identified by the participants.

Socio-economic status is a common barrier in many circumstances [19, 20]. This leads to patients being pulled back when it comes to seeking medical attention. This also impacts of other factors such as medication, Similar studies have found that affordability of medications has been the major barrier followed by the amount of medications, compliance of patients, inadequate time with patients, patient knowledge and education accessories, and the skill of physicians to deal with the dietary plans and enhancing patient adherence and compliance [14, 21–23]. Limited time with patients, inaccessibility of risk calculators and technology, choice of tools and guidelines to use, physicians trust and confidence in the guidelines, integration of calculators in clinic settings with proper instruction on flow of use, knowledge and training on risk calculators, adequate staffing and retaining of trained staff in the system, a clinical advocate or team leader to reinforce the guidelines, communication with the team on the guidelines and the variations in various calculators are also some of the barriers. A similar study identified barriers such as inadequate time, issues with guidelines, communication, roles of heath care givers, inadequate knowledge and experience and attitudes of personnel [24, 25]. The fears of patients on the potential side-effects or the consumption of medications, inadequate evidences of use among patients, the expenses of medication and willingness to obtain those medication and poor insight of their conditions are also potential barriers [26, 27]. Other studies stated that guidelines are too time consuming while some said they are not beneficial at all [28, 29]. Some barriers with WHO adapted guidelines include poor knowledge and familiarity on CVD prevention and the guidelines and lack of agreement to the guideline approaches, attitude-based included lack of motivation and communication and coordination issues with team members, behavior-based, that is patient factors and environmental-based factors such as lack of time due to work schedules, lack of resources, organizational constraints, and poor record system [30].

Effective communication and re-enforcement of advice on health related behavior is paramount as highlighted by the participants. Strategies such as use of educational materials, involvement of family in decision making, use of translators in vernacular language have been suggested by the physicians. They also indicate that patients should be engaged in formulating their management plans as in this way patients will feel encouraged and take ownership of their health thus, compliance to the agreed plan will be high. Other studies also support that the role of communication is essential in prevention and reduction of CVDs. It is suggested that the combination of internet-based reporting system and clinics encouraged better communication with tele-medicine system viewed as cost and time efficient as compared to regular visits to doctors [31]. A review supported WHO stated that Mobile Health or sending messages through texts could be a potential tool for interventions in LMIC [32]. The use of color-coding charts and absolute risk calculators to demonstrate risks and effects of risk factors and pictures to demonstrate the visual images also play an important role [33]. In addition, shared-decision making process enables and encourages positive attitude towards behavior change [34]. This could be potentially utilized to deal with the associated social stigma that has been highlighted by the physicians. This will encourage and build confidence in patients.

This study participants are of the view that in order to standardize practice of CVD risk management, policies need to be centrally coordinated. Having a special set of qualified people to develop and evaluate the cardiovascular policies implementation for the whole country would be vital. Participants believe that collaboration amongst doctors will help improve service delivery. Similarly, an article published on the integrated team approach to the care of the patient with CVD demonstrated that guideline-based prevention is effective in controlling and preventing CVDs. These approaches are executed a multi-disciplinary team that is guided by a physician during the phases of care [35].

Poor access to specialized care has been highlighted by all physicians in this study. The participants’ believe that process of obtaining specialized care is lengthy and in most cases the clinical picture of patients has significantly changed from the time patient was initially referred and the time patient actually gets seen by the specialist. This delay is most times due to poor coordination of patient movement and lack of resources. A review of the current process of patient movement for specialist review and care has been suggested by the participants’ of this study. A study in Brazil highlighted that access barriers like transportation, cost and distance were some of the barriers patients were not able to use cardiac rehabilitation programs [19].

Physicians generally stated that all existing laws and legislations in regards to CVDs are needed to be reinforced by the relevant authorities. Some stated that stricter policies are needed to discourage people from high risk behaviours, for example, a complete ban on cigarette smoking. This has been also highlighted in a similar study in Brazil [19]. Moreover, a background paper on “Health and & Non-Communicable Diseases” had stated that significant gaps were identified in the implementation of tobacco-control recommendations in Pacific islands. The paper indicated that the Pacific Island countries need to increase the tobacco prices together with an increase in excise duties to 70% of the retail cigarette costs. It was also stated that these countries should advocate, prosecute and implement the prevailing laws and regulations if they aim to achieve the declared “Tobacco Free Pacific” by the year 2025 [10]. Moreover, a review paper was done on the role of law and governance reform where the need for a comprehensive approach to NCDs risk factors, improving accessibility of treatment and addressing the burden of the illness was highlighted. The prevention and treatment priority demonstrated that legal and regulatory modifications fall at the core of an efficacious national response to NCDs. The most effective approach to impact the burden of diseases in LMICs in the “best buys” approach. In addition, it is important to incorporate leadership and accountability and implementation of effective policies and laws as part of the efforts [36]. It is also suggested that policies catalyze and integrate regulatory, legislative and multi-sectoral actions that would allow a conducive environment in appropriate health sectors [37].

Research and studies play an important role in improving patient care and service delivery. Most of the participants were satisfied with the current studies and evidences, however there is a general consensus that the current evidence is not being translated into clinical practice and more effort is needed by all stakeholders to ensure the evidence materializes into practice in order to ensure improvement in CVD risk management. A similar study evaluated the physicians’ perception and attitude towards evidence-based medicine and practice found that the physicians attained articulated positive attitudes towards evidence-based medicine however their competency was retained at moderate level [38]. Research makes better physicians with its capability of critically evaluating advanced evidences and providing the best of the care to patients [23, 39]. A physician-researcher would have the ability to illustrate high quality safety of patients and quality enhancement research in their peculiar practices in the future. This serves as an encouragement for physicians to involve in research and studies [39].

Literature suggests that policy-makers require the best presented evidence and expansion in the role of researchers and practitioners and engage the problem, policy and politics aspects to implement evidence-based policy development, advance effective specific policy elements and institute improvement, expansion or termination of policies depending on the outcome [40].

### Limitations

This study had some limitations. Participants of this study may not be a reflection of the entire physician population in the country. It would be valuable to obtain perceptions of all other disciplines who are involved as a multi-disciplinary team to deal with CVDs and its consequences. There were challenges found during data collection due to the current global pandemic, COVID-19. Also face-to-face interview would have been more suitable and comprehensive in conducting in-depth interviews. The perceptions of physicians in a rural setting may have differed due to limited resources and patient, physician and health setting factors. With the recordings, it is also possible the participants would have given ideal answers and restricted their actual views and opinions.

## Conclusion

It can be concluded that perceptions of physicians is ideal to acknowledge the gaps in CVD risk management. Some of the barriers identified include patient, physician and health system factors. The patient factors include education level, insight of condition, myths and beliefs, socio-economic support, financial status, access to health care, compliance, medications, social stigma and self-motivation. Physician factors include education and knowledge, experience, preference, attitude, availability of resources, trainings, adherence to guidelines and self-motivation and confidence. The health system factors include patient-doctor ratio, availability of allied health care workers, medication factors, investigations and tests required, multi-disciplinary approach, health promotion materials and strategies, unforeseen circumstances such as COVID-19 pandemic and patient-record systems.

These challenges hinder a successful and goal-oriented health system to perform and produce quality health service delivery to its full potential. Medical advancements has been made to improve clinical medicine, thus, it is expressively important to keep up with those advancements through CMEs, monitoring and evaluation and continues research and studies. Some of the recommendations for the primary care physicians include uplifting self-motivation, strengthening personal abilities, and engaging in continuous medical education to keep up with the current CVD risk management by WHO and medical evidences. The recommendations for the Ministry includes addition of the tools and guidelines in the medical school and internship curriculum, involving all medical officers in the SOPD settings, encouraging training and workshops for physicians, encouraging peer-support groups for physicians and strengthening the current protocols in place. The recommendations for future researchers include focusing on the multi-disciplines involved in CVD risk management, study in all settings and including private sectors perceptions as well.

## Data Availability

The datasets generated and/or analysed during the current study are not publicly available due to privacy or ethical restrictions but are available from the corresponding author on reasonable request.

## Abbreviations

CVD: Cardiovascular diseases
PCP: Primary care physicians
PHC: Primary health care
SOPD: Special outpatients department
GOPD: General outpatients department
MDG: Millennium development goals
WHO: World health organization
SDG: Sustainable development goals
HC: Health center
MOIC: Medical office in-charge
COVID-19: Coronavirus disease of 2019
CME: Continuous medical education
LMIC: Low- and middle-income countries
FNU: Fiji National University
CHREC: College Health Research Ethics Committee
DMO: Divisional medical officer
MohMS: Ministry of Health and Medical Services
MBBS: Bachelor of Medicine and Bachelor of Surgery
